# Variability and homogeneity of cardiovascular magnetic resonance myocardial T2-mapping in volunteers compared to patients with edema

**DOI:** 10.1186/1532-429X-15-27

**Published:** 2013-03-27

**Authors:** Ralf Wassmuth, Marcel Prothmann, Wolfgang Utz, Matthias Dieringer, Florian von Knobelsdorff-Brenkenhoff, Andreas Greiser, Jeanette Schulz-Menger

**Affiliations:** 1 Department of Cardiology and Nephrology, Working Group Cardiac MRI, Humboldt University Berlin, Charite Campus Buch Experimental and Clinical Research Center and HELIOS Klinikum Berlin Buch, Berlin, Germany; 2 Siemens AG Healthcare Sector, Erlangen, Germany

## Abstract

**Background:**

The aim of the study was to test the reproducibility and variability of myocardial T2 mapping in relation to sequence type and spatial orientation in a large group of healthy volunteers. For control T2 mapping was also applied in patients with true edema. Cardiovascular magnetic resonance (CMR) T2-mapping has potential for the detection and quantification of myocardial edema. Clinical experience is limited so far. The variability and potential pitfalls in broad application are unknown.

**Methods:**

Healthy volunteers (n = 73, 35 ± 13 years) and patients with edema (n = 28, 55 ± 17 years) underwent CMR at 1.5 T. Steady state free precession (SSFP) cine loops and T2-weighted spin echo images were obtained. In patients, additionally late gadolinium enhancement images were acquired. We obtained T2 maps in midventricular short axis (SAX) and four-chamber view (4CV) based on images with T2 preparation times of 0, 24, 55 ms and compared fast low angle shot (FLASH) and SSFP readout. 10 volunteers were scanned twice on separate days. Two observers analysed segmental and global T2 per slice.

**Results:**

In volunteers global myocardial T2 systematically differed depending on image orientation and sequence (FLASH 52 ± 5 vs. SSFP 55 ± 5 ms in SAX and 57 ± 6 vs. 59 ± 6 ms in 4CV; p < 0.0001 for both). Anteroseptal and apical segments had higher T2 than inferior and basal segments (SAX: 59 ± 6 vs. 48 ± 5 ms for FLASH and 59 ± 7 vs. 52 ± 4 ms for SSFP; p < 0.0001 for both). 14 volunteers had segments with T2 ≥ 70 ms. Mean intraobserver variability was 1.07 ± 1.03 ms (r = 0.94); interobserver variability was 1.6 ± 1.5 ms (r = 0.87). The coefficient of variation for repeated scans was 7.6% for SAX and 6.6% for 4CV. Mapping revealed focally increased T2 (73 ± 9 vs. 51 ± 3 ms in remote myocardium; p < 0.0001) in all patients with edema.

**Conclusions:**

Myocardial T2 mapping is technically feasible and highly reproducible. It can detect focal edema und differentiate it from normal myocardium. Increased T2 was found in some volunteers most likely due to partial volume and residual motion.

## Background

In acute myocardial infarction or inflammation T2-weighted cardiovascular magnetic resonance (CMR) can detect myocardial edema in vivo [1-4]. Increased myocardial water content changes magnetic relaxation properties that influence the CMR signal [5,6]. This can be clinically helpful to differentiate acute from chronic myocardial lesions [7,8] and to detect even small acute myocardial damage very early [9,10]. T2-weighted short tau triple inversion recovery fast spin echo (STIR) can result in pronounced contrast between bright edema and hypointense normal myocardium [11]. However, T2-weighted imaging may suffer from signal loss in higher heart rates and arrhythmias as well as imperfect blood suppression in areas of slow blood flow hampering delineation of edema [12-14]. Therefore alternatives for more stable detection of edema and easier quantification are clinically warranted [15].

CMR T2-mapping is a promising tool for characterizing myocardial edema [16-19]. While initial reports focused on the depiction of focal lesions the aim of this study was to assess the variability of myocardial T2 relaxation times in volunteers and patients and the influence of sequence type, spatial orientation and spatial resolution.

## Methods

The local ethical committee approved the study. All participants were enrolled after informed consent was obtained.

The study complies with the declaration of Helsinki. The ethical committee of Charite Medical University approved the study on January 27th, 2011. The application number was EA1/276/10.

### Volunteers

We scanned 73 healthy volunteers (13 female, 20–70 years, mean 35 ± 13 years, median 30 years, BMI 23 ± 3 kg/m2) without any cardiovascular disease, no symptoms of inflammation and a normal electrocardiogram. All participants were seen by a cardiologist. We discouraged alcohol intake one day before the scan to avoid inflammatory reaction [20]. Ten volunteers were scanned twice (time delay 469 ± 219 days, median 381 days) to assess interstudy variability.

### Patients

We investigated a group of consecutive patients (n = 28; 8 females, age 55 ± 17 years, range 20–81 years) with acute myocardial damage. Edema was defined as a regional area of hyperintense signal on T2-weighted fast spin echo images corresponding to evidence of focal myocardial damage like wall motion abnormality or late gadolinium enhancement (LGE). The group comprises 20 patients with acute myocardial infarction (imaging 3 ± 1 days after admission), 5 patients with acute myocarditis (four male, median age 22 years, positive troponin and typical LGE lesions in all of them, CMR on 1 ± 1 day after admission), 2 postmenopausal female patients with Takotsubo cardiomyopathy (typical presentation and history, no scar, but transient apical ballooning) and 1 patient with cardiac sarcoidosis (acute admission with positive troponin, focal edema corresponding to typical LGE lesion).

### CMR

Using a 1.5 T scanner (Magnetom Avanto, Siemens Erlangen, Germany, software version B17) with a 12-channel chest coil we obtained steady state free precession (SSFP) cine loops (repetition time 2.8 ms, echo time 1.2 ms, slice thickness 8 mm, flip angle 80 degrees, in-plane resolution 1.8 mm/pixel) during breathhold in three long and at least one midventricular short axis matching the slice position for mapping. T2-weighted STIR images (repetition time = 2 RR-intervals, echo time 58 ms, slice thickness 8 mm, in-plane resolution 1.3 × 1.3 mm/pixel, imaging in mid-diastole) were obtained in the same short axis as cine and additionally in long axis, if a focal abnormality was seen. In patients we additionally acquired fast low angle shot (FLASH) inversion recovery gradient echo LGE images in short and long axes (slice thickness 8 mm, in-plane resolution 1.8 × 1.4 mm/pixel) after 0.2 mmol/kg Gadolinium-DTPA.

**Figure 1:**
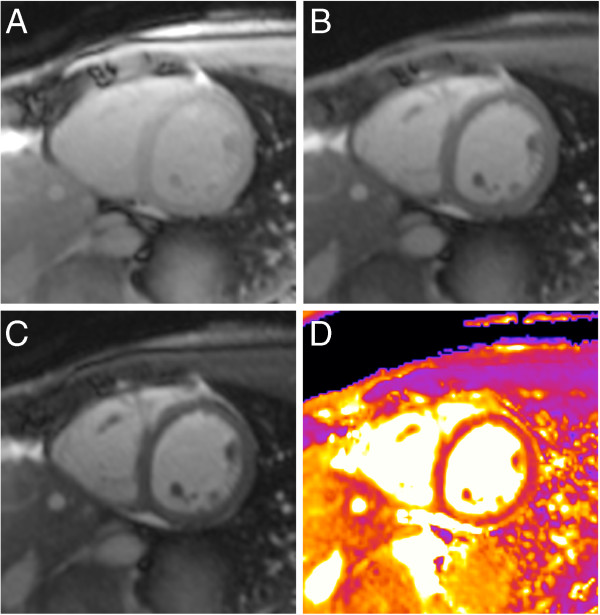
**Raw data and resulting T2-map.** T2 mapping in a healthy volunteer in midventricular short axis orientation. The color-coded map (**D**) is generated after motion correction and based on three images (**A-C**) with different T2 preparation times (**A**: 0 ms, **B**: 24 ms, **C**: 55 ms).

For T2-mapping we applied a prototype sequence based on either FLASH or SSFP gradient echo [16] in one midventricular short axis (SAX) and four-chamber-view (4CV) matching the orientation of cine and STIR images. Slice thickness was 8 mm for all images. The pixel-wise map was based on three single shot images with preceding T2 preparation pulses employing T2 evolution times of 0, 24 and 55 ms (Figure 1). Read-out trajectory was centric for FLASH and linear for SSFP. A non-rigid motion correction was applied to reduce in-plane motion artefacts [17]. Pixel-wise curve fitting was done automatically as part of the inline imaging processing. Spatial resolution was 2.7 × 2.1 mm/pixel. Scan time was 12 heartbeats. The maximum acquisition window was 150 ms per single-shot image. Mid-diastole was chosen for image acquisition. In a subgroup of 24 volunteers additional SSFP-based maps with higher spatial resolution of 2.2 × 1.8 mm/pixel were acquired during the same scan. In patients image orientation was adjusted to the focal abnormality, e.g. in Takotsubo cardiomyopathy mapping was done in long axis. In 14/28 patients mapping data were acquired with the SSFP-based sequence only.

### Phantom

We scanned a spherical phantom of 20 cm diameter filled with manganese chloride doped distilled water. The RR-interval was simulated to be 1000 ms. A 2D multi contrast spin echo sequence (repetition time = 2000 ms, 32 echo times equally spaced from 7 ms to 224 ms) in conjunction with a mono-exponential three-parameter-fit served as T2 reference measurement [21]. We additionally obtained measurements using the FLASH and SSFP sequences with identical parameters as used for imaging volunteers and patients.

### Data analysis

For signal analysis we used Osirix (version 3.9.1 http://www.osirix-viewer.com) and QMASS (version 7, Medis, Leiden, The Netherlands) in all subjects. The endocardial and epicardial contours were manually drawn on the last corresponding T2-weighted raw image with the echo time of 55 ms. The myocardium was then segmented (manually in Osirix, automatically in QMass) into 6 segments according to the AHA segmentation scheme [22]. Contours were copied to the map, corrected when necessary and global and segmental T2-values were recorded. Two independent observers analysed all the volunteer data. Both do have considerable experience in CMR image analysis (> 15 years and > 3 years, respectively) and put much effort in avoiding inclusion of blood or fat while drawing regions of interest. Interstudy reproducibility was measured for SAX and 4CV. The coefficient of variation (CoV) was calculated as the ratio of the standard deviation of the interscan difference divided by the mean of the measurement. Anteroseptal enddiastolic myocardial wall thickness was measured on short axis and four-chamber-view cine frames and compared to T2-times in the same segment. To assess residual diastolic wall motion M-mode-like myocardial signal intensity projections over time were generated from 2D SSFP short axis and four-chamber-view cine images in selected patients using an in-house developed implementation in Matlab 7.1 (The Mathworks, Natick, MA).

The same investigator analyzed all SSFP volunteer maps twice. In patients areas of focal abnormality matching hyperintense signal in STIR and LGE were selected and compared to remote myocardium. We excluded hypointense infarction cores indicating microvascular obstruction [17].

Data are given as mean ± standard deviation unless indicated otherwise. We compared results from different sequences and orientations and repeated scans with paired student´s t-test and Pearson correlation coefficient r. P-values < 0.05 were considered significant. 95% confidence intervals (CI) were calculated. Bland-Altman-plots were obtained to analyse intra- and interobserver variability. Volunteer and patient data were compared using 95% tolerance intervals with 90% coverage. These tolerance intervals cover 90% of all observations of a normal distribution with 95% confidence when the mean and the standard deviation are known. Influencing factors were identified by sequence using the candidates age, gender, heart rate, body surface area, body mass index and the interaction terms in an analysis of variance with forward selection (criterion for selection p < 5%). We compared the data on healthy volunteers and patients using a mixed linear model with compound symmetry as working correlation matrix to account for the multiple measurements of up to four sequences by subject. Gender and age (35 years or younger, more than 35 years) were included as covariates. We performed a multivariate analysis including wall thickness, diastolic motion and a combination thereof to assess the impact on myocardial T2.

## Results

### Phantom

**Figure 2:**
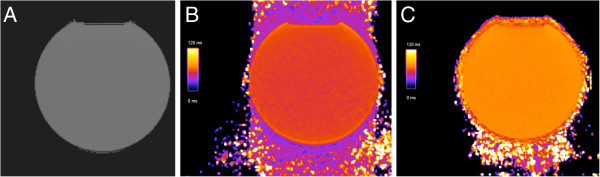
**T2 measurements in a phantom.** A multiecho spin echo sequence as a gold standard (**A**) resulted in T2 = 62.0 ± 0.5, FLASH T2 map (**B**) yielded T2 = 55.5 ± 1.0 ms and SSFP T2 map (**C**) resulted in T2 = 70.9 ± 0.8 ms.

Evaluation of multi contrast spin echo data in the phantom yielded T2 = 62.0 ± 0.5 ms. The T2-mapping variant using FLASH readout resulted in T2 = 55.5 ± 1.0 ms, whereas SSFP readout revealed T2 = 70.9 ± 0.8 ms (Figure 2).

### Volunteers

Four volunteers were excluded from analysis due to pathological findings (pleural effusions, tachycardic atrial fibrillation and left ventricular hypertrophy). The remaining 69 formed the healthy study group.

### Global T2 measurements in volunteers

**Figure 3:**
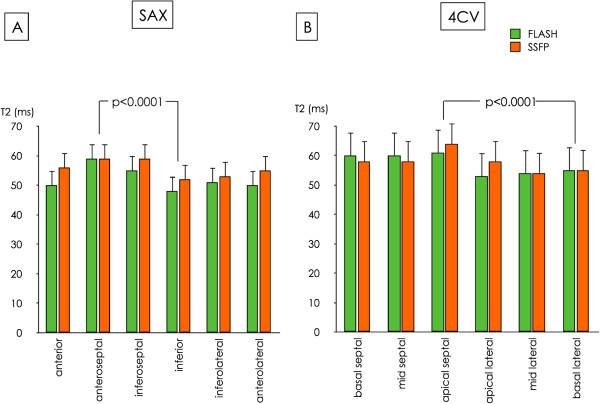
**Spatial variation of T2 in volunteers.** Segmental measurements reveal spatial variation of myocardial T2 in short axis (SAX) (**A**) and four-chamber view (4CV) (**B**). The Y-axis shows T2-times in ms. Note higher T2-values in anteroseptal and apical septal segments compared to inferior and basal septal segments.

In all subjects maps could be generated in diagnostic quality. Mean heart rate was 73 ± 10 bpm. Global myocardial T2 measurements are summarized in Table 1. Global T2-values did not correlate with heart rate (r = 0.002), age (r = 0.30), body surface area (r = 0.44) or body mass index (r = 0.36). SSFP-based mapping resulted in higher values for T2 than FLASH-based mapping (SAX: 55 ± 5 vs. 52 ± 5 ms; 4CV: 59 ± 6 vs. 57 ± 6 ms; p < 0.0001 for both). This was true for global (Table 1) and segmental measurements (Figure 3). Global T2-mapping resulted in higher values in 4CV-orientation than in short axis (p < 0.0001 for both sequences; Table 1)*.*

**Table 1:** Global myocardial T2 relaxation times (ms) in volunteers

		**FLASH**	**SSFP**	
**SAX**	Mean	52 ± 5	55 ± 5	p < 0.0001
	Range	41–62	46–69	
	CI (5–95%)	51–53	54–57	
**4CV**	Mean	57 ± 6	59 ± 6	p < 0.0001
	Range	46–74	51–80	
	CI (5–95%)	56–59	57–60	
		p < 0.0001	p < 0.0001	

CI denotes confidence interval, *SAX* = short axis, *4CV* = four-chamber view, *FLASH* = fast low angle shot, *SSFP* = steady state free precession.

### Segmental T2 measurements in volunteers

With both sequences anteroseptal segments had higher T2-values than inferior segments in SAX (59 ± 6 vs. 48 ± 5 ms for FLASH and 59 ± 7 vs. 52 ± 4 ms for SSFP; p < 0.0001 for both, Figure 3). In 4CV the apical septal segment had higher T2 than the basal lateral segment for both FLASH (61 ± 8 vs. 55 ± 6 ms; p < 0.0001) and SSFP (64 ± 9 vs. 55 ± 6 ms; p < 0.0001; Figure 3). In SAX the mean absolute difference between a single segment and the whole slice was 4 ± 1 ms and 3 ± 2 ms for FLASH and SSFP, respectively. In 4CV the mean absolute difference between a single segment and a global measurement was 5 ± 2 ms for both, FLASH and SSFP.

### Variability in volunteers

**Figure 4:**
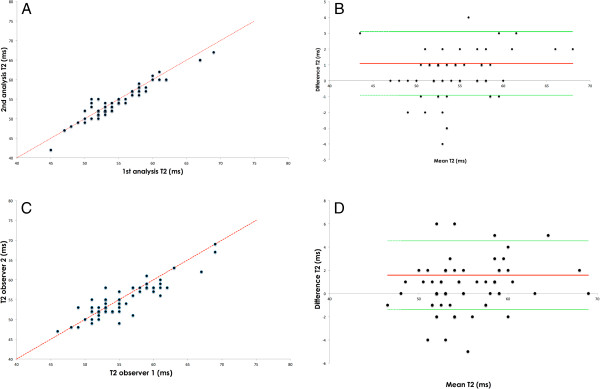
**Intra- and interobserver variability in volunteers.** Intra- (**A**) and (**B**) and interobserver (**C**) and (**D**) variability for global myocardial T2 in short axis based on the steady state free precession sequence in volunteers. Scatterplots (**A** and **C**) and Bland-Altman plots (**B**) and (**D**) are given. Red lines indicate bias. Green lines indicate the limits of agreement (1.96 x standard deviation). Due to overlap dots stand for more than 1 data point.

Intra- and inter-observer variability was low (Figure 4). The mean difference for T2 between repeated measurements in one observer was 1.07 ± 1.03 ms (r = 0.94). Mean difference for T2 between two observers was 1.6 ± 1.5 ms (r = 0.87). The CoV for repeated scans was 7.6% for SAX and 6.6% for 4CV. The results for myocardial T2 did not differ depending on the analysis software used.

### Outliers among volunteers

**Figure 5:**
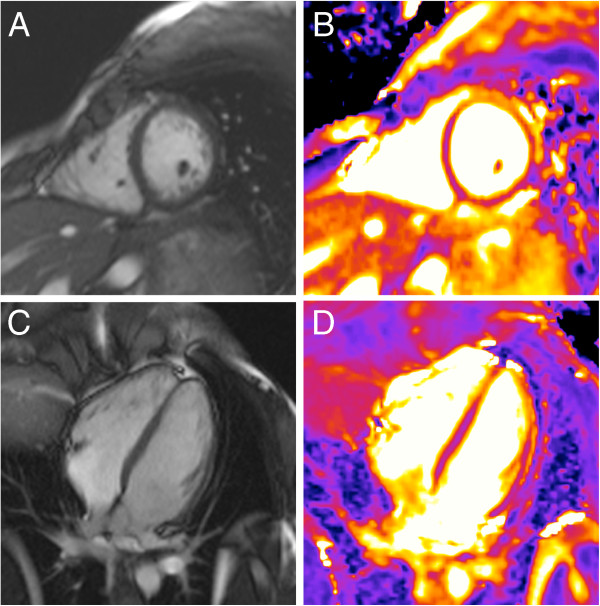
**A healthy 22-year-old female volunteer with unusually high T2.** Diastolic short axis (**A**) and four-chamber view (**C**) cine frames illustrate thin anterior and apical walls. T2-maps in short axis (**B**) and four-chamber view (**D**) reveal markedly elevated T2 in anterior and apical segments. Global myocardial T2 in short axis was 69 ms, anterior T2 was 77 ms.

**Figure 6:**
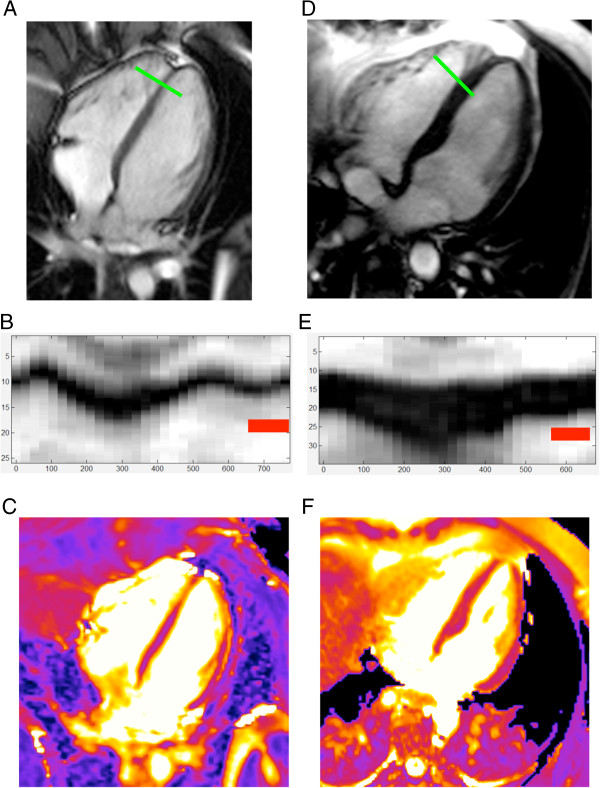
**Wall thickness and diastolic motion influence myocardial T2.** A young female volunteer has an apical septal wall thickness of 2 mm (**A**). In m-mode projections (**B**) and (**E**) the y-axis indicates distance in mm, the x-axis indicates time within the cardiac cycle in ms. The red bar represents the acquisition window. In this case M-mode projection reveals residual diastolic motion of 2.8 mm (**B**). Apical septal T2 was 80 ms (**C**). A 46-year-old male volunteer of had an apical-septal wall thickness of 7 mm (**D**), a diastolic wall motion of 1.3 mm (**E**) and a segmental T2 of 55 ms (**F**).

Even among the normal volunteer group we found segmental and global T2-values equal to or higher than 70 ms (Figure 5) in 14 of 69 healthy subjects. On a segmental basis this occurred in 3/414 segments in FLASH and 7/414 segments in SSFP in short axis. In 4CV maps it occurred in 26/414 segments in FLASH and 29/414 in SSFP. In 13 out of 14 volunteers the apical septal segment was affected. In 4CV there were 4 volunteers each, who had global myocardial T2 ≥70 ms in FLASH or SSFP. There was no significant difference in age between those with and without T2 ≥70 ms (31 ± 12 vs. 38 ± 13 years; p = 0.08). Anteroseptal wall thickness as measured in enddiastolic cine frames (mean 3.5 mm, range 2–8 mm for 4CV) was inversely related to myocardial T2. The thinner the anteroseptal segment was, the higher was T2 (r = −0.6 for 4CV). The m-mode analysis revealed a mean residual diastolic motion of 2.3 mm (range 0.1 – 5.2 mm). More diastolic motion correlated with higher myocardial T2 (r = 0.37). In a multivariate analysis diastolic wall motion (p < 0.001) and the combination of wall thickness and motion (p < 0.003) significantly correlated with myocardial T2. Dichotomizing the volunteer group for age and gender reveals the larger range for myocardial T2 in younger and mainly female volunteers (Figure 6).

**Figure 7:**
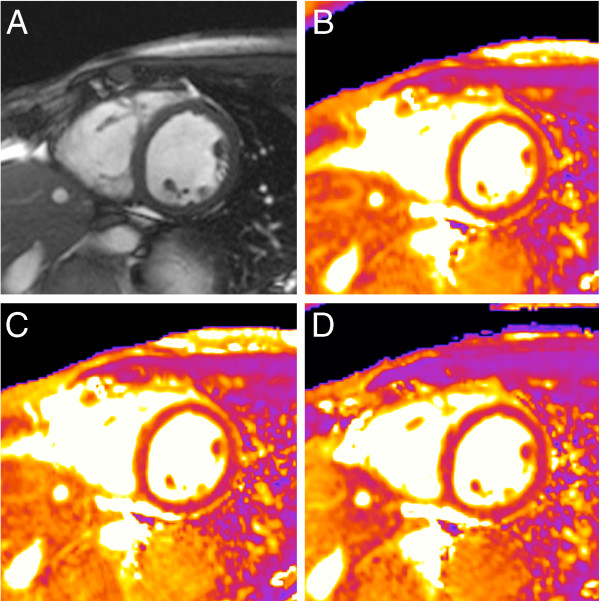
**Influence of spatial resolution.** T2-mapping in a healthy 43-year-old male with different spatial resolution. Corresponding cine frame in (**A**). While the map with an initial spatial resolution of 2.7 × 2.1 mm/pixel (**B**) suggests spots of increased T2 in the anteroseptal segment, these spots diminish with increasing spatial resolution to 2.2 × 1.8 mm/pixel (**C**) and improved motion correction on top (**D**). The acquisition window was 130 ms in (**D**). Anteroseptal T2 was 62 ms in (**B**), 58 ms in (**C**) and 51 ms in (**D**).

Mapping with improved spatial resolution did change results in individual subjects (Figure 7), but not for the whole group (p = 0.3) Even after improving spatial resolution 8 out of 24 volunteers had segmental myocardial T2 ≥70 ms on 4CV maps, while only one segment was affected in SAX. In 4CV this occurred in the apical septal segment in all but one case.

### Measurements in patients

**Figure 8:**
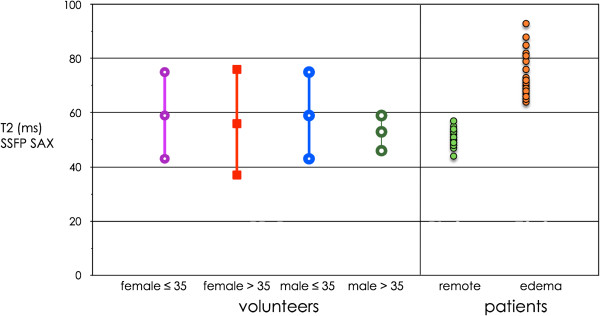
**Comparison of global myocardial T2 in healthy volunteers (left) and focal edema in patients (right).** Data are shown for SSFP-based mapping in short axis. Of note, in controls the bars do not represent mean ± standard deviation, but mean ± 95% tolerance interval with 90% coverage. For patients, individual data points are shown.

**Figure 9:**
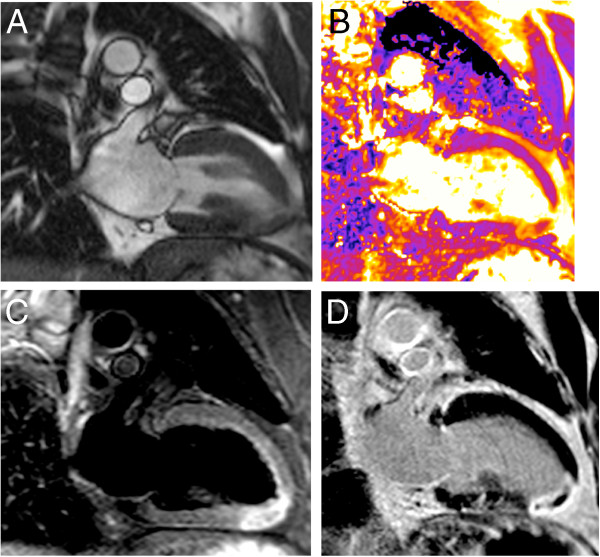
**T2-mapping in acute myocardial infarction.** CMR in a 47-year-old male patient 2 days after apical inferior infarction. The distal left anterior descending artery was occluded. All images shown here are in two-chamber-orientation. (**A**): systolic cine frame reveals apical-inferior wall motion abnormality. (**B**): T2-map shows an apical area of increased T2 reflected by orange color with a centre of lower T2 indicated by pink colour. (**C**): conventional T2-weighted STIR image shows focal area of hyperintense signal with a hypointense core. (**D**): Late gadolinium enhancement shows myocardial scar with core of microvascular obstruction.

**Figure 10:**
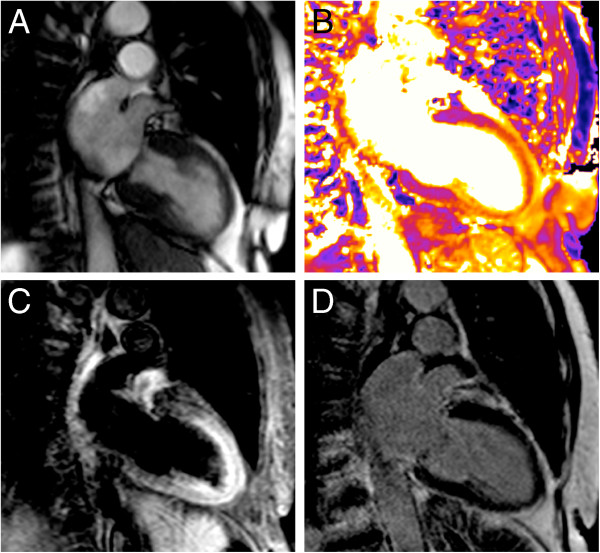
**T2-mapping in Takotsubo cardiomyopathy.** 81-year-old female presented with Takotsubo cardiomyopathy including transient ECG-changes and elevated troponin after a generalized seizure. CMR was done 3 days after the initial event. All images shown here are in two-chamber-orientation. Systolic cine frame reveals apical ballooning (**A**). T2 map indicates elevated apical myocardial T2 reflected by orange colour (**B**). Conventional T2-weighted STIR image shows increased myocardial signal intensity in the apex that might be difficult to differentiate from intraluminal blood signal in the case of slow flow (**C**). Late gadolinium enhancement excludes myocardial scar (**D**).

In all 28 patients with acute myocardial damage areas of increased T2 could be detected. Using SSFP-based mapping T2 of edema was 73 ± 9 ms on average (range 64–99 ms, 95% CI 70–76 ms), while T2 of remote myocardium was 51 ± 3 ms (range 44–57 ms, 95% CI 50–52 ms; Figure 8). T2 did not differ between ischemic (Figure 9) and nonischemic (Figure 10) edema.

## Discussion

T2-mapping is technically feasible with low intra-, interobserver and inter-scan variability and does not depend on heart rate. Overall our mapping results with mean T2 around 55 ms for normal myocardium match previous experiences [6,16,23-27].

As far as T2 mapping in patients with acute myocardial damage is concerned we can confirm earlier small patient studies [17,19]. In patients with acute myocardial infarction or inflammation T2-mapping detects focal edema and reveals increased myocardial T2-values with small variability. Our results for remote myocardium nicely match those for our elderly male volunteers.

Therefore it appears straightforward to characterize a clearly delineated pathologic lesion in clinical routine. However, it might be more challenging to exclude any lesion in a subject with no or mild disease.

Our systematic analysis in a large group of volunteers revealed, that SSFP-based T2-mapping resulted in slightly higher values than FLASH. In early phantom studies as well as our own experiment (Figure 2) mapping with FLASH showed good agreement with “true” T2 values [28]. Therefore SSFP tends to overestimate true T2 as demonstrated before [16,27]. On the other hand SSFP-based mapping offers more signal to noise [27] and suffers less from image artefacts than FLASH due to centric readout of FLASH-based mapping [16].

Partial volume and motion effects most likely explain higher global T2 in 4CV than in SAX as well as spatial inhomogeneity with higher T2 in anterior and anteroseptal segments. Wall thickness (i.e. thin walls) and residual diastolic motion were related to higher myocardial T2. Close observation of cine loops and analysis with m-mode-like projection confirm diastolic wall motion in particular in young volunteers with thin walls [29]. The low intra- and interobserver variability indicates that these outliers are not simply a problem of suboptimal contouring.

Inclusion of even small amounts of neighbouring entities like blood (T2 of about 200 ms) and pericardial fat (T2 of about 80 ms) can grossly influence the measurement of myocardial T2. Improving spatial resolution should minimize partial-volume effect, however this also prolongs the acquisition window increasing motion artefacts. We found T2-values of up to 80 ms even in our volunteer group, where as the T2 for edema associated with acute infarction has been reported to be 69 ± 6 ms [17]. Therefore care has to be taken not to generate false-positive findings of edema in subjects with thin and highly mobile walls. In patients with true edema this is less a problem due to increased wall thickness in the presence of edema [30].

For the future imaging at individually optimized time points within the cardiac cycle should be considered if cine imaging indicates abundant myocardial motion even in mid-diastole. Depending on individual wall motion across the cardiac cycle in cine loops systole or early diastole may be chosen. However, previous T1 mapping experiments in volunteers indicate that results might slightly differ between systole and diastole [31].

**Figure 11:**
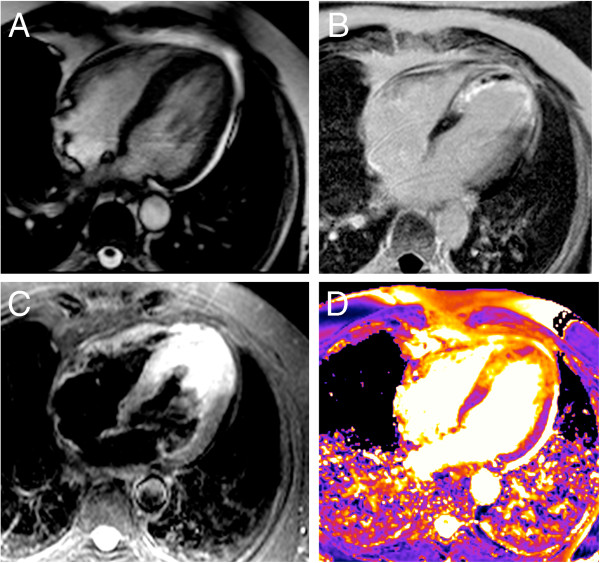
**T2-mapping in acute apical infarction.** The diastolic cine frame (**A**) reveals apical septal wall thickening in the area of edema. Late gadolinium enhancement shows apical infarction scar with microvascular obstruction (**B**). While conventional T2-weighted STIR image depicts both, edema and bright slow flowing blood within the lumen (**C**), T2 map correctly indicates edema color-coded in orange with an apical T2 of 91 ms (**D**). The microvascular obstruction corresponds to a T2 of 60 ms, color-coded in pink. Basal T2 was 54 ms.

A clinical advantage for myocardial T2 mapping can be expected in particular in patients with pronounced apical wall motion abnormalities where conventional T2-weighted imaging can be challenging. A severe apical wall motion abnormality may result in hyperintense intraluminal blood signal that is difficult to differentiate from bright signal within the wall that truly reflects edema. In these circumstances T2-mapping seems to offer additional options for clinical routine to confirm true edema while conventional T2-weighted spin echo remains inconclusive (Figure 11). In addition the breathhold time of the mapping sequence is by far shorter than that of a conventional fast spin echo sequence, which makes imaging easier for a heart failure patient. Conventional T2-weighted imaging is semi-quantitative at best requiring a reference structure. T2-mapping may open the way for a truly quantitative approach in assessing acute damage.

We enrolled patients with focal lesions only. Theoretically mapping offers the potential to quantify diffuse tissue damage that is less obvious on conventional CMR images. Future studies including patients with known diffuse disease might enlighten this phenomenon. We included two cases of typical Takotsubo cardiomyopathy and one case of sarcoidosis with typical late Gadolinium enhancement corresponding to focal edema. Edema has been previously described in both entities including a large multicentre study for Takotsubo cardiomyopathy [32-35].

### Limitations

The power calculation took into account the differentiation of patients and volunteers as a whole group. The study and its sample size were not designed to reveal subtle differences among e.g. women of different decades of age. There is no dedicated gold standard for true edema. We assumed edema in myocardial areas where it made sense clinically based on concomitant late gadolinium enhancement or obvious wall motion abnormalities in patients with evidence of acute myocardial damage in laboratory results and electrocardiogram.

## Conclusions

Myocardial T2 mapping is technically feasible and highly reproducible in a large number of normal volunteers. Differences in sequences and spatial resolution result in small differences in myocardial T2 values. Quantifying T2 easily detects focal myocardial edema and differentiates it from remote myocardium. T2 mapping may offer a more stable and truly quantitative alternative for edema detection in cases when conventional T2-weighted imaging fails. Mapping in thin and rapidly moving myocardial walls can result in overestimation of myocardial T2 and must not be confused with true edema.

## Abbreviations

CMR: Cardiovascular magnetic resonance; STIR: Short tau triple inversion recovery; SSFP: Steady state free precession; FLASH: Fast low angle shot; SAX: Short axis; 4CV: Four-chamber view; CI: Confidence interval; LGE: Late gadolinium enhancement

## Competing interests

A Greiser is an employee of Siemens AG, Erlangen, Germany. The authors declare no other competing interests.

## Authors’ contributions

RW and JSM conceived of and designed the study. RW, MP, WU, MD, AG and FVK acquired, analysed and interpreted the data. RW wrote the manuscript with input from WU, FVK, MD and JSM. All authors read and approved the final manuscript.
